# Selective Regional Loss of Cortical Synapses Lacking Presynaptic Mitochondria in the 5xFAD Mouse Model

**DOI:** 10.3389/fnana.2021.690168

**Published:** 2021-06-25

**Authors:** Na-young Seo, Gyu Hyun Kim, Jeong Eun Noh, Ji Won Shin, Chan Hee Lee, Kea Joo Lee

**Affiliations:** ^1^Neural Circuits Research Group, Korea Brain Research Institute, Daegu, South Korea; ^2^Department of Brain and Cognitive Sciences, Daegu Gyeongbuk Institute of Science and Technology, Daegu, South Korea

**Keywords:** Alzheimer's disease, 5xFAD, synapse, mitochondria, cortex, electron microscopy

## Abstract

Synaptic loss in Alzheimer's disease (AD) is strongly correlated with cognitive impairment. Accumulating evidence indicates that amyloid pathology leads to synaptic degeneration and mitochondrial damage in AD. However, it remains unclear whether synapses and presynaptic mitochondria are differentially affected in various cortical regions of the AD brain at the ultrastructural level. Using serial block-face scanning electron microscopy, we assessed synaptic structures in the medial prefrontal cortex (mPFC) and primary visual cortex (V1) of the 5xFAD mouse model of AD. At 6 months of age, 5xFAD mice exhibited significantly elevated levels of amyloid deposition in layer 2/3 of the mPFC but not V1. Accordingly, three-dimensional reconstruction of synaptic connectivity revealed a significant reduction in excitatory synaptic density in layer 2 of the mPFC, but not V1, of male transgenic mice. Notably, the density of synapses lacking presynaptic mitochondria was selectively decreased in the mPFC of 5xFAD mice, with no change in the density of mitochondria-containing synapses. Further classification of spines into shape categories confirmed a preferential loss of thin spines whose presynaptic boutons were largely devoid of mitochondria in the 5xFAD mPFC. Furthermore, the number of mitochondria per bouton in spared mitochondria-containing boutons was reduced in the mPFC, but not V1, of 5xFAD mice. Collectively, these results highlight region-specific vulnerability of cortical synapses to amyloid deposition and suggest that the presence of presynaptic mitochondria may affect synaptic degeneration in AD.

## Introduction

Alzheimer's disease (AD) is the most common form of dementia, and effective treatments to halt its progression are lacking. The key pathological hallmarks of AD include extracellular amyloid beta (Aβ) plaques and intracellular neurofibrillary tau tangles (Hyman and Tanzi, 1992; Serrano-Pozo et al., 2011). It is well-established that AD patients and animal models present with synaptic and neuronal loss, brain atrophy, neuroinflammation, and cognitive dysfunction (Mega et al., 1996; Krstic and Knuesel, 2013; DeTure and Dickson, 2019). Among these neuropathological alterations, reduced synaptic density is the most strongly correlated with cognitive decline in AD (DeKosky and Scheff, 1990; Scheff et al., 2007), indicating that synaptic dysfunction preceding plaque and tangle pathology underpins behavioral impairments in AD (Oddo et al., 2003). Accumulating evidence indicates that amyloid deposition or Aβ oligomers surrounding dense plaques are highly toxic and induce synapse loss and cognitive impairment (Walsh et al., 2002; Cleary et al., 2005; Hsieh et al., 2006; Shankar et al., 2007). Synaptic abnormalities are particularly evident near amyloid plaques (Spires et al., 2005; Spires-Jones et al., 2007). Furthermore, Aβ-induced synapse loss can be prevented in AD mouse models by administrating Aβ-targeting antibody or silencing Aβ-induced synaptic proteins involved in synaptic degeneration (Lee et al., 2019; Jang et al., 2020; Xiao et al., 2020), suggesting a causal relationship between amyloid pathology and synaptic loss.

In addition to synapse loss, defects in synaptic mitochondria have been proposed as key contributors to synaptic degeneration in AD. In general, less than half of synapses possess mitochondria in their presynaptic boutons (Shepherd and Harris, 1998), and mitochondria-containing boutons are more stable than boutons without mitochondria (Lees et al., 2019). Due to their proximal location to synaptic vesicles, presynaptic mitochondria modulate synaptic transmission via local calcium homeostasis and ATP production (Smith et al., 2016; Devine and Kittler, 2018). Therefore, disruption to synaptic mitochondria or their trafficking to synaptic boutons may induce synaptic failure and subsequent removal (Pickett et al., 2018). Several studies have reported that Aβ aggregation affects the structural and functional properties of synaptic mitochondria and their synaptic transport in brain tissues of AD patients and amyloid precursor protein (APP) transgenic mice (Mungarro-Menchaca et al., 2002; Caspersen et al., 2005; Manczak et al., 2006, 2018; Pickett et al., 2018). Moreover, the administration of Aβ-targeting monoclonal antibody alleviates Aβ-induced mitochondrial damage in an AD mouse model (Xiao et al., 2020), suggesting a causative role of amyloid pathology in mitochondrial dysfunction. It remains largely unknown how synaptic mitochondria are associated with Aβ-mediated synapse loss observed in AD.

Topographically, amyloid plaques gradually spread throughout the cortex (Masters et al., 2015). Although the pattern of pathogenic Aβ propagation is less predictable, amyloid deposits are predominantly observed in the frontal and temporal association cortices (DeTure and Dickson, 2019). In contrast, primary sensory, motor, and visual cortical areas tend to be less affected in the early stages of AD compared to the association cortex (Arnold et al., 1991; Braak and Braak, 1991). Light microscopic studies have confirmed the differential cortical and subcortical distribution of amyloid plaques in AD murine models (Gail Canter et al., 2019; Whitesell et al., 2019). However, it remains unclear whether synapses and presynaptic mitochondria are differentially affected in cortical regions of the AD mouse brain according to the amyloid distribution at the ultrastructural level. Therefore, this study aimed to dissect ultrastructural differences in synapses and presynaptic mitochondria in different regions of the AD mouse brain. We employed three-dimensional synaptic analysis using serial block-face scanning electron microscopy (SB-SEM) in the medial prefrontal cortex (mPFC) and primary visual cortex (V1) of 5xFAD mice, a well-established AD murine model, to explore cortical region-specific or synapse type-specific differences in synapse loss. We focused on layer 2 pyramidal neurons because they receive diverse corticocortical inputs to integrate information across cortical areas (Luo et al., 2017), and structural synaptic plasticity is well-established in this layer (Holtmaat and Svoboda, 2009). We hypothesize that synapses in different cortical areas of the AD mouse brain will be affected differentially depending on the levels of amyloid deposition.

## Materials and Methods

### Animals

5xFAD mice (Tg6799, MMRRC Stock No: 34840-JAX, MGI: 3693208) were maintained as hemizygous on a C57BL/6 background. All mice were maintained on a 12 h light/dark cycle in the animal facility of the Korea Brain Research Institute (KBRI). All animal experiments were conducted in accordance with the NIH Guidelines for Care and Use of Laboratory Animals and approved by the Institutional Animal Care and Use Committee (IACUC-19-00033 and IACUC-20-00040).

### Immunohistochemistry

Six-month-old 5xFAD and wild-type (WT) mice (*n* = 5 per group; three males and two females) were anesthetized with isoflurane and perfused transcardially with 0.1 M phosphate-buffered saline (PBS). The brains were dissected, immersed in 4% paraformaldehyde for 48 h at 4°C, and cryoprotected in 30% sucrose in PBS at 4°C. After freezing the brains, 40 μm-thick coronal sections were obtained using a cryostat. Sections were permeabilized for 2 h in a PBS solution containing 1% TritonX-100 and 10% normal donkey serum, followed by incubation with mouse anti-6E10 antibody (BioLegend, Cat #803002, PRID: AB_2564654) in a PBS solution containing 0.2% TritonX-100 and 2% normal donkey serum overnight at 4°C. The following day, sections were washed with PBS and incubated with Alexa 555-conjugated donkey anti-mouse antibody (*Invitrogen*, Cat #A-31570, PRID: AB_2536180) for 2 h at room temperature. Hoechst 33342 (Sigma-Aldrich, Cat #B2261) was used for nuclear staining. Sections containing mPFC and V1 were mounted on glass slides, and images were captured using a digital slide scanner (3D Histech). Images were acquired with 20x/NA 0.8 objective, resolution of 0.32 μm/pixel, and image size of 1858 x 1057 pixels. The region of interest was manually selected in images stained with Hoechst 33342 (total area analyzed per mouse: mPFC 0.39 ± 0.009, V1 0.24 ± 0.005 mm^2^). The number of puncta per unit area and the puncta size of amyloid deposits were quantified in the thresholded images using ImageJ software (NIH). In each region, the puncta size and density values of two sections per each animal were averaged to produce an animal mean. The bregma coordinates of the sections were as follows: mPFC (2.22- and 0.98-mm anterior to bregma) and V1 (2.54- and 3.52-mm posterior to bregma).

### Electron Microscopy and Analysis

Male mice (*n* = 3 per group) were perfused with 2% paraformaldehyde and 2.5% glutaraldehyde in 0.15 M cacodylate buffer (pH 7.4). Brains were collected, and 150 μm-thick sections were dissected into the prelimbic mPFC and V1. Slices were post-fixed in 2% OsO4/1.5% potassium ferrocyanide for 1 h. Samples were then immersed in 1% thiocarbohydrazide (Sigma-Aldrich, Cat #223220) in ddH_2_O for 20 min followed by 2% OsO4 for 30 min. Tissues were incubated in 1% uranyl acetate at 4°C overnight and a lead aspartate solution at 60°C for 30 min. Samples were serially dehydrated in ethanol followed by acetone and subsequently embedded in 7% (w/v) conductive Epon 812 resin (EMS, Cat #14120) mixed with Ketjen black powder, as previously described (Nguyen et al., 2016). Tissue blocks were imaged with a Merlin VP field-emission scanning electron microscope (SEM; Carl Zeiss) equipped with 3View2 (Gatan). Images were acquired with a 30 μm aperture, high vacuum, voltage of 1.5 kV, image size of 5,000 × 5,000 pixels, and x–y resolution of 8 nm at a nominal thickness of 50 nm. Two stacks of 200 serial images of cortical layer 2 were obtained per mouse (32,000 μm^3^ per mouse), one each of the mPFC and V1. Image stacks were acquired in plaque-free regions to observe widespread loss of spines and synapses in 5xFAD mice, although spine loss was more prominent within 20 μm of the plaque edge (Spires et al., 2005). Images were processed using ImageJ (NIH) and Fiji plugins (http://fiji.sc/wiki/index.php/Fiji). TrakEM2 was used to assemble images for reconstruction. We randomly selected spiny dendritic segments with lengths ranging from 12 to 15 μm. To ensure that our analysis was restricted to pyramidal neurons, we avoided dendritic segments with a few or no spines. In total, 24 dendritic branches were manually segmented in the mPFC (12 dendrites per group) and 18 in the V1 (9 dendrites per group) using reconstruction software (https://synapseweb.clm.utexas.edu/) by three annotators blinded to genotype. The densities of spines, synapses with or without mitochondria, and synapses with multiple mitochondria were quantified per 10 μm of dendritic segment. We restricted our analyses to synapses formed on dendritic spines, the postsynaptic components of most excitatory glutamatergic synapses in the central nervous system (Hering and Sheng, 2001). As structural correlates of synaptic strength, spine volume including the head and neck, and axon-spine interface (ASI), the surface of direct contact between axonal boutons and spines were measured due to easier identification of exact ASI borders compared to the postsynaptic density (PSD) in SB-SEM images (de Vivo et al., 2017). For classification of spine shape, protrusions were categorized as thin, mushroom, stubby, branched, or filopodia (Harris et al., 1992). Spine volume and maximum diameter of the spine head and neck were measured for spine shape categorization using the 3ds max software (Autodesk). Briefly, spines under 1 μm in length without a constricted neck were defined as stubby. Spines with a head diameter two-fold larger than their neck diameter were classified as mushroom. Spines with two or more heads were defined as branched. Protrusions longer than 3 μm without presynaptic partners were defined as filopodia. The rest of them were classified as thin. In addition, the maximal and minimal diameter of each dendritic shaft were measured to produce a regularity index for dendritic thickness by dividing the minimal diameter with the maximal diameter. All quantified parameters in this study are listed in Supplementary Table 1.

### Statistics

All data are presented as mean ± SEM. Statistical analyses were performed using GraphPad Prism software (RRID: SCR_002798). Density analyses of SB-SEM data involve one measurement per dendrite. After D'Agostino–Pearson or Shapiro–Wilk normality test, normally distributed data were compared using an unpaired Student's *t*-test or a two-way analysis of variance (ANOVA) with a *post-hoc* Tukey test. Non-normally distributed data were compared by a Mann–Whitney U test or a Kruskal–Wallis test with a *post-hoc* Dunn's multiple comparison. Differences were considered statistically significant at *P* < 0.05.

## Results

### Regional Differences in Amyloid Accumulation in the 5xFAD Cortex

**Figure 1:**
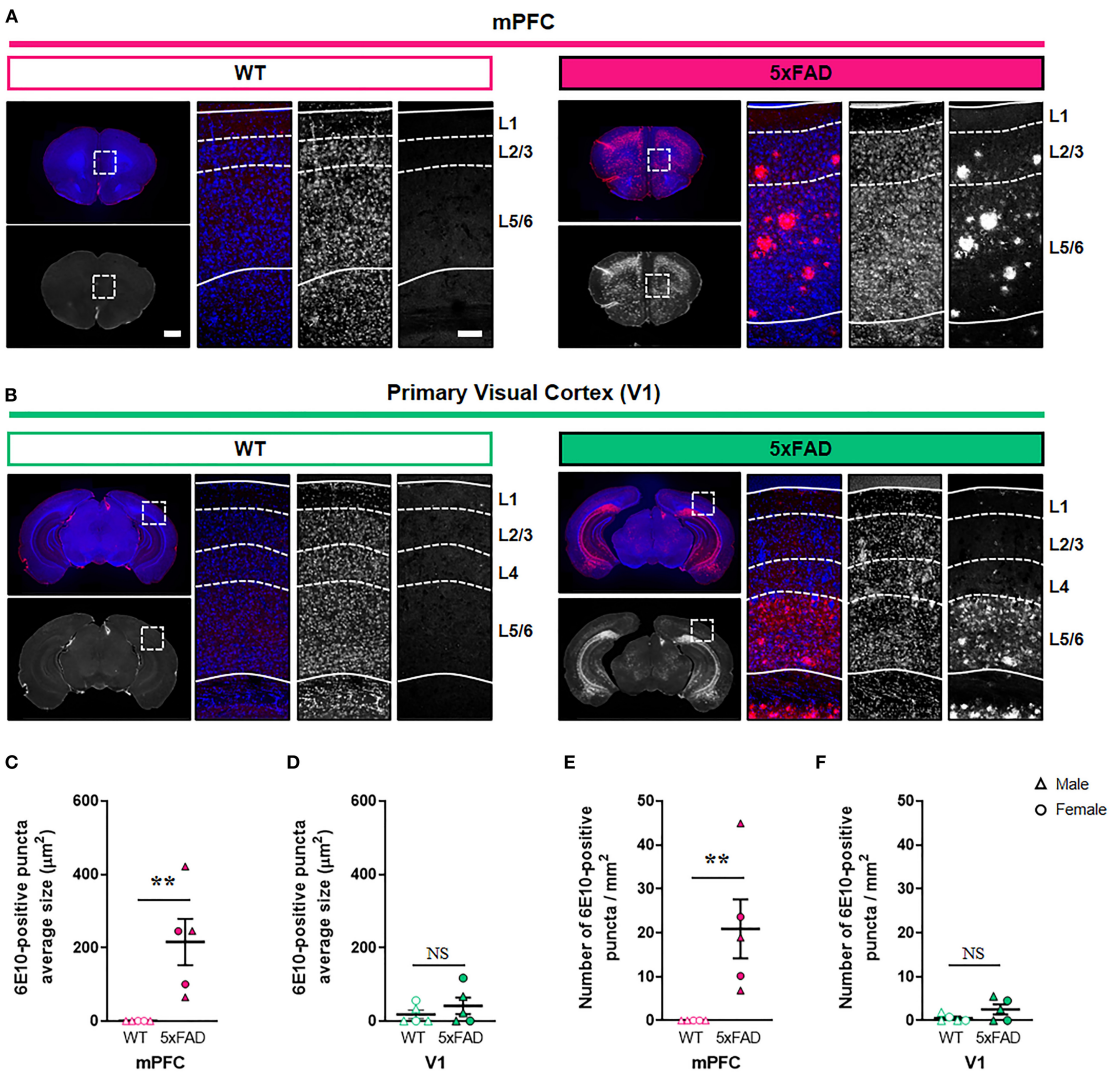
Elevated amyloid deposition in the mPFC, but not V1, of 6-month-old 5xFAD mice. **(A,B)** Representative fluorescence images of amyloid plaques stained with 6E10 antibody in the mPFC and V1 of wild-type (WT) and 5xFAD mice. Dashed boxes in whole brain images indicate regions of interest. Dotted lines indicate quantified cortical layers. mPFC, medial prefrontal cortex; V1, primary visual cortex. Scale bars, 1 mm (low magnification) and 100 μm (high magnification). **(C,D)** Quantification of amyloid puncta size in layer 2/3 of the mPFC (C) and V1 (D), respectively (n= 5 animals per group). **(E,F)** Quantification of amyloid puncta density in layer 2/3 of the mPFC **(E)** and V1 **(F)**, respectively (*n* = 5 animals per group). **P < 0.01; Mann-Whitney U test.

During the early stages of AD, Aβ accumulates predominantly in the association cortex including mPFC, whereas V1 is relatively spared (Arnold et al., 1991; Braak and Braak, 1991; DeTure and Dickson, 2019). To test whether the region-specific difference in amyloid deposition exists in the cortex of AD murine model, we compared the levels of amyloid deposits in the mPFC and V1 between 6-month-old 5xFAD and WT mice in a cortical layer-specific manner (Figure 1; Supplementary Table 1; Supplementary Figure 1). The 5xFAD is one of the most widely used mouse models of AD and expresses five familial AD-linked mutations (three in human *APP* and two in human *PSEN1*) (Oakley et al., 2006). At 6 months of age, these transgenic mice exhibit neuropathological phenotypes, including extracellular amyloid deposition in the cortex, abnormal synaptic transmission, decreased synaptic marker protein expression, and cognitive impairments (Oakley et al., 2006; Crowe and Ellis-Davies, 2013; Richard et al., 2015). Nevertheless, spatiotemporal patterns of synapse loss have not been investigated. Immunohistochemistry with an anti-Aβ 6E10 antibody revealed a significant elevation in both the amyloid puncta size and the puncta density in layer 2/3 of the 5xFAD mPFC compared to WT mPFC (Figures 1A–C,E) (puncta size, *P* = 0.008; density, *P* = 0.008; Mann-Whitney U test). In contrast, no significant difference in amyloid deposition in layer 2/3 of V1 was found between genotypes (puncta size, *P* = 0.52; density, *P* =0.29; Mann-Whitney U test) (Figures 1D,F). In layer 1, the amyloid puncta size and density was also heightened in the 5xFAD mPFC, but not V1, between groups (Supplementary Figures 1A–E). While the mPFC lacks a detectable layer 4 (Uylings et al., 2003), the amyloid deposition was modestly but significantly more pronounced in this layer of the 5xFAD V1 compared to WT V1 (Supplementary Figures 1E,F). In layer 5/6, the amyloid puncta size and density was significantly increased in both the mPFC and V1 of 5xFAD mice (Supplementary Figures 1H–K). Together, these results demonstrate a cortical layer-specific differences in amyloid pathology between WT and 5xFAD mice at 6 months of age.

### Selective Loss of Synapses Lacking Presynaptic Mitochondria in the 5xFAD mPFC

**Figure 2:**
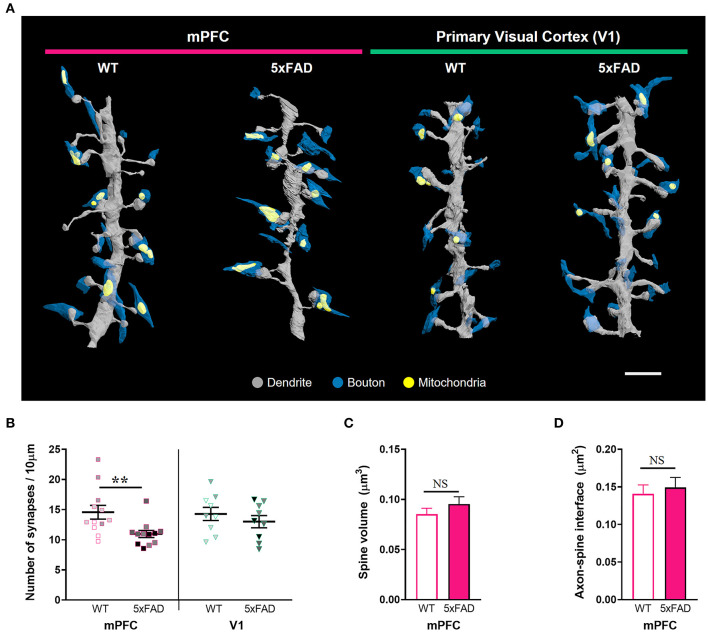
Synapse loss in the mPFC, but not V1, of 6-month-old 5xFAD mice. **(A)** 3D reconstruction of SB-SEM images showing representative dendritic segments in the mPFC and V1 of WT and 5xFAD mice. Gray, dendrite; blue, presynaptic boutons; yellow, mitochondria. Scale bar, 2 μm. **(B)** Quantification of synapse density in the mPFC and V1 of WT and 5xFAD mice, respectively (*n* = 12 dendrites for mPFC and 9 dendrites for V1 per group). **P < 0.01; Kruskal–Wallis test with *post-hoc* Dunn test. NS, not significant. The data points from each animal were displayed in the same color. **(C,D)** Quantification of average spine volume (**C**, *n* = 228–289 spines per group) and ASI area (**D**, *n* = 188–251 synapses per group) in the mPFC of WT and 5xFAD mice. NS, not significant. Mann-Whitney U test.

Given the substantial amyloid accumulation in layer 2/3 of the 5xFAD mPFC but not V1, we performed a three-dimensional analysis of excitatory synaptic number and structure in the layer 2 of the mPFC and V1 of 5xFAD and WT mice using SB-SEM (Figure 2; Supplementary Figure 2). The density of synapses on dendritic segments was significantly lower in the mPFC, but not in the V1, of 5xFAD mice compared to WT animals (Figures 2A,B; Supplementary Table 1) (mPFC, *P* = 0.009; V1, *P* = 0.42; Kruskal–Wallis test with *post-hoc* Dunn test), implying region-specific vulnerability of cortical synapses to amyloid deposition in the 5xFAD mice. Despite synapse loss, individual spine volume and synaptic apposition (ASI) area of the remaining synapses in the 5xFAD mPFC were comparable to those in the WT mPFC (Figures 2C,D) (spine volume, *P* = 0.62; ASI area, *P* = 0.18; Mann-Whitney U test). These results are consistent with previous reports revealing no difference in synaptic size in the AD cortex and hippocampus (West et al., 2009; Pickett et al., 2018; Dominguez-Alvaro et al., 2019), although reduced synaptic size was also observed in the hippocampal CA1 area of AD patients (Montero-Crespo et al., 2021).

**Figure 3:**
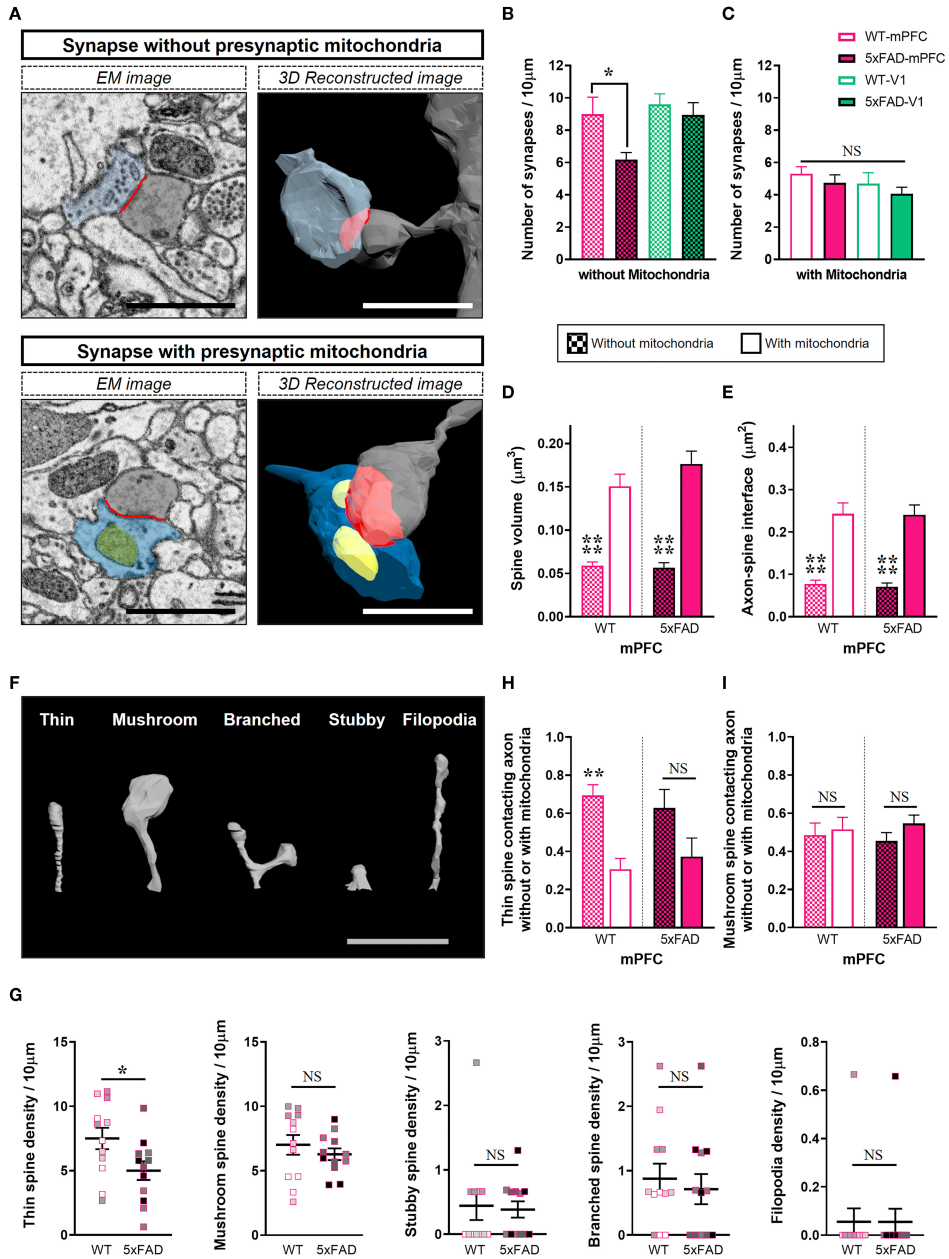
Selective loss of synapses lacking presynaptic mitochondria in the 5xFAD mPFC. **(A)** Representative EM (left) and 3D reconstructed (right) images of synapses either containing or lacking presynaptic mitochondria. Blue, presynaptic bouton; yellow, mitochondria; gray, dendritic spine; red, axon-spine interface (ASI). Scale bar, 1 μm. **(B,C)** Quantification of densities of synapses without **(B)** or with **(C)** presynaptic mitochondria in the mPFC and V1 of WT and 5xFAD mice (*n* = 9–12 dendrites per group). *P < 0.05; two-way ANOVA with *post-hoc* Tukey test. **(D,E)** Quantification of average spine volume **(D)** and ASI area **(E)** of synapses with or without presynaptic mitochondria (*n* = 87–95 mitochondria-containing synapses; 100–154 mitochondria-lacking synapses). ****P < 0.0001; Kruskal–Wallis test with *post-hoc* Dunn test. **(F)** Representative image of spine shape categories. **(G)** Quantification of the number of spines according to shape per 10 μm dendritic length (*n* = 9–12 dendrites per group). *P < 0.05; unpaired *t*-test for thin, mushroom, and branched spines; Mann-Whitney U test for stubby and filopodia. The data points from each animal were displayed in the same color. **(H,I)** The proportion of thin spines **(H)** and mushroom spines **(I)** contacting boutons with or without mitochondria in each dendritic segment (n=9–12 dendrites per group). **P < 0.01; NS, not significant; two-way ANOVA with *post-hoc* Tukey test.

Considering that mitochondria in presynaptic boutons provide calcium buffering capacity and energy for modulation of synaptic activity, we conjectured that mitochondria-containing synapses would be more resistant to Aβ-induced synapse elimination in the 5xFAD mPFC. To test this hypothesis, synapses were distinguished by the presence or absence of presynaptic mitochondria (Figure 3; Supplementary Table 1). Notably, we observed a selective reduction in the density of synapses without presynaptic mitochondria in the 5xFAD mPFC but not V1 (Figures 3A,B), with no change in the density of mitochondria-containing synapses between genotypes and region (Figure 3C) (density of synapses lacking presynaptic mitochondria: mPFC, *P* = 0.045; V1, *P* = 0.95; density of synapses with presynaptic mitochondria: mPFC, *P* = 0.84; V1, *P* = 0.85; two-way ANOVA with *post-hoc* Tukey test). Mitochondria-containing synapses exhibited significantly larger spine volume and ASI size compared to mitochondria-devoid synapses, regardless of genotype or region (Figures 3D,E; Supplementary Figures 3A,B) (*P* < 0.0001; Kruskal–Wallis test with *post-hoc* Dunn test). To corroborate these findings, we classified 3D reconstructed spines into five shape categories: thin, mushroom, stubby, branched, or filopodia. Thin spines are more dynamic and less stable than mushroom spines (Bourne and Harris, 2007). We observed that 5xFAD mice had fewer thin-type spines in the mPFC compared to WT animals (Figures 3F,G; Supplementary Table 1) (thin, *P* = 0.03; mushroom, *P* = 0.42; branched, *P* = 0.63; unpaired *t*-test; stubby, *P* = 0.84; filopodia, *P* > 0.99; Mann-Whitney U test). In contrast, no significant between-group differences were observed in the distribution of spine shapes in V1 (Supplementary Figure 3C). The proportions of thin spines contacting boutons lacking mitochondria were significantly higher than those of thin spines contacting mitochondria-containing boutons regardless of genotype or region, with the exception of the 5xFAD mPFC (Figure 3H; Supplementary Figure 3D) (WT, *P* = 0.008; 5xFAD, *P* = 0.12; two-way ANOVA with *post-hoc* Tukey test), indicating the preferential loss of thin spines without presynaptic mitochondria in the 5xFAD mPFC. The proportions of mushroom spines contacting boutons with or without mitochondria were comparable regardless of genotype or region (Figure 3I; Supplementary Figure 3E) (WT, *P* = 0.98; 5xFAD, *P* = 0.63; two-way ANOVA with *post-hoc* Tukey test).

### Reduced Number of Boutons Containing Multiple Mitochondria in the 5xFAD mPFC

**Figure 4:**
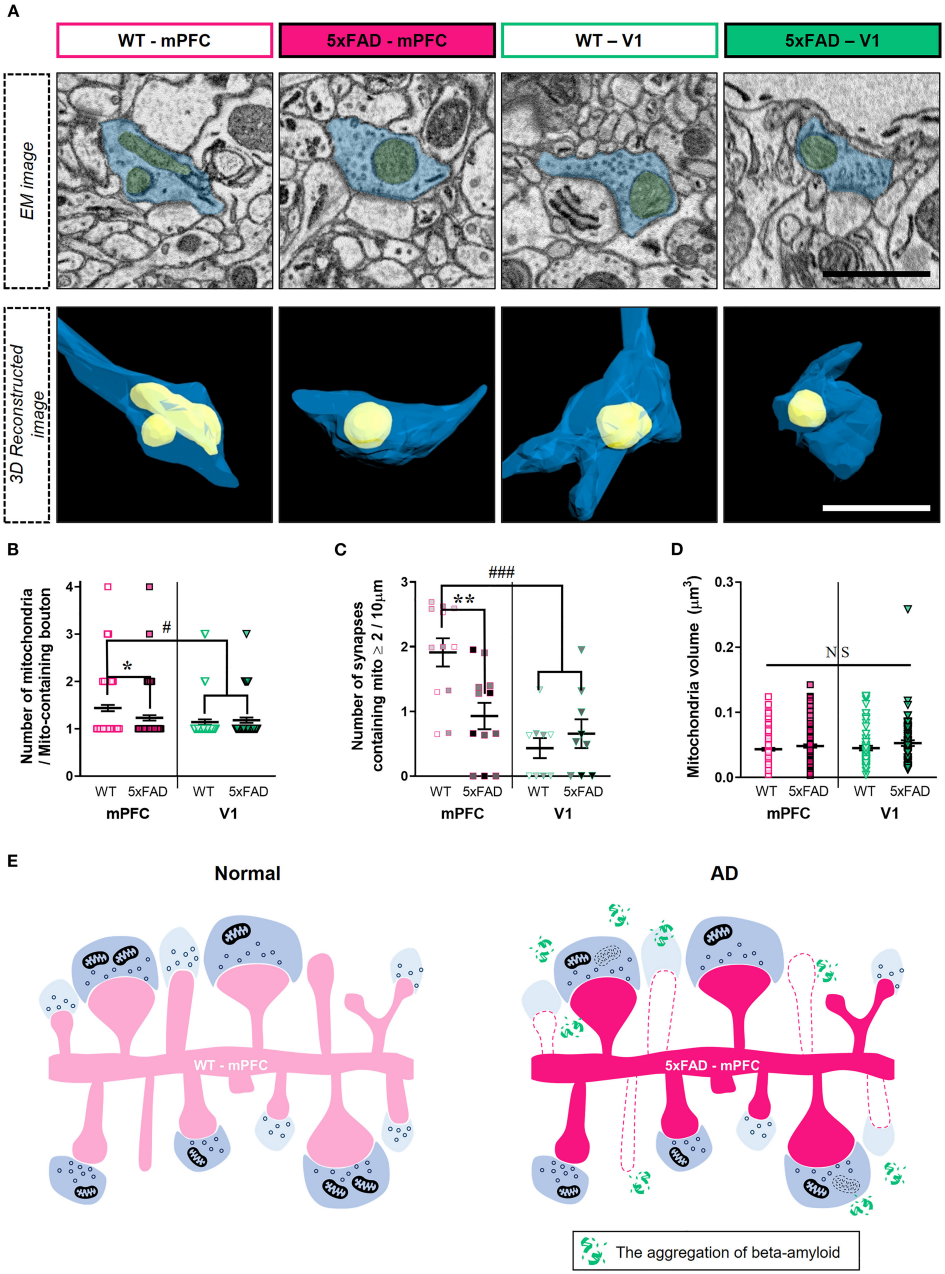
Reduced number of boutons containing multiple-mitochondria in the 5xFAD mPFC. **(A)** Representative EM (top) and reconstructed (bottom) images of presynaptic boutons containing mitochondria in the mPFC and V1 of WT and 5xFAD mice. Blue, presynaptic bouton; yellow, mitochondria. **(B)** Quantification of the number of presynaptic mitochondria per bouton (*n* = 61–96 boutons per group). *P < 0.05, #*P* < 0.05; Kruskal–Wallis test with *post-hoc* Dunn test. **(C)** The density of synapses containing multiple mitochondria (*n* = 9–12 dendrites from 3 animals per group). **P < 0.01, ###*P* < 0.001; two-way ANOVA with *post-hoc* Tukey test. The data points from each animal were displayed in the same color. **(D)** Average volume of individual mitochondria (*n* = 71–138 per group). *P* > 0.38; Kruskal–Wallis test with *post-hoc* Dunn test. **(E)** Schematic model for synaptic alterations in the 5xFAD mPFC.

Although the number of synapses lacking presynaptic mitochondria was selectively reduced in the 5xFAD mPFC (Figure 3), amyloid deposition may also affect mitochondria-containing synapses. Thus, we quantified the number of mitochondria per presynaptic bouton (Figure 4; Supplementary Table 1). We observed lower numbers of mitochondria per bouton in the mPFC between 5xFAD and WT mice (Figures 4A,B) (mPFC, *P* = 0.03; V1, *P* > 0.99; Kruskal–Wallis test with *post-hoc* Dunn test). Among mitochondria-containing boutons, the number of boutons with multiple mitochondria was significantly lower in the 5xFAD mPFC, but not V1 (Figure 4C) (mPFC, *P* = 0.005; V1, *P* = 0.89; two-way ANOVA with *post-hoc* Tukey test). Notably, the number of mitochondria per boutons and the density of synapses involving multiple mitochondria in the mPFC of WT mice were significantly higher compared to V1 of both WT and 5xFAD animals (Figures 4B,C) (mitochondria #/bouton: WT-mPFC vs. WT-V1, *P* = 0.0004; WT-mPFC vs. 5xFAD-V1, *P* = 0.02; Kruskal–Wallis test with *post-hoc* Dunn test; synaptic density with ≥ 2 mitochondria: WT-mPFC vs. WT-V1, *P* < 0.0001; WT-mPFC vs. 5xFAD-V1, *P* = 0.0007; two-way ANOVA with *post-hoc* Tukey test), suggesting a cortical region-specific difference in presynaptic mitochondrial availability. No significant differences were noted in mitochondrial volume between groups or regions (Figure 4D) (*P* > 0.38; Kruskal–Wallis test with *post-hoc* Dunn test).

## Discussion

Amyloid pathology induces synapse loss and cognitive dysfunction in AD (DeKosky and Scheff, 1990; Walsh et al., 2002; Oddo et al., 2003; Cleary et al., 2005; Jang et al., 2020). A multiphoton microscopy study also revealed that a loss of cortical dendritic spines adjacent to amyloid plaques resulted from enhanced spine elimination, with no change in spine formation in an AD mouse model expressing a mutant form of human APP (Spires-Jones et al., 2007). Due to the progressive spread of amyloid plaques through the brain, synapses in different cortical areas can be affected gradually depending on the levels of amyloid deposition. In this study, we identified significantly elevated amyloid accumulation in the mPFC but not V1 of 5xFAD mice at 6 months of age (Figure 1), in support of previous observations (DeTure and Dickson, 2019; Gail Canter et al., 2019). Despite the transgene expression driven by the Thy-1 promotor, it remains intriguing that there are region-specific differences in amyloid deposition in this mouse line. In accordance with the differential cortical amyloid distribution, three-dimensional synaptic analysis using SB-SEM revealed lower excitatory synaptic density in layer 2 of the mPFC, but not in V1, of 5xFAD mice (Figure 2). Why is the mPFC more vulnerable to amyloid accumulation? One possibility may be tied to the high levels of baseline neural activity (Buckner et al., 2005). Indeed, chronic upregulation of neural activity promotes Aβ concentrations and plaque deposition (Yuan and Grutzendler, 2016), whereas reduced Aβ generation with a β-secretase inhibitor prevents hyperactivity in the frontal cortex (Keskin et al., 2017). This potential mechanism fits well within the context of our observed phenotype since the mPFC is a crucial component of the default-mode network that is most active at a resting state (Whitfield-Gabrieli and Ford, 2012). Additionally, the default-mode network is known to be substantially affected by amyloid pathology in AD patients (Buckner et al., 2005). Layer 2 pyramidal neurons in the mPFC are innervated by inputs from the midline thalamic nucleus, basolateral amygdala, ventral hippocampus, and contralateral mPFC (Gabbott et al., 2005; Vertes, 2006; Little and Carter, 2012), while V1 receives main visual inputs from the lateral geniculate nucleus of the thalamus (Guido, 2018). Since our imaging volume and method do not allow for the differentiation of long-distance inputs, it is important to assess if synaptic loss in the mPFC is due to input-specific abnormalities using viral tracing of circuit connectivity or array tomography to label specific inputs. It would be also interesting to investigate potential changes in symmetric (inhibitory) synapses in the 5xFAD mPFC in follow-up studies.

In the 5xFAD mPFC, we observed a selective decrease in density of synapses lacking presynaptic mitochondria and of thin spines contacting mitochondria-lacking boutons (Figure 3). Although we did not observe a difference in the density of mitochondria-containing synapses between genotypes, the number of mitochondria per bouton and number of boutons containing multiple mitochondria were lower in the 5xFAD mPFC (Figure 4), consistent with a previous report on the reduced boutons with multiple mitochondria in the cortex of AD patients (Pickett et al., 2018). Notably, the WT mPFC had significantly more mitochondria per bouton and higher density of boutons containing multiple mitochondria compared to V1 of both WT and 5xFAD animals (Figure 4), pointing to a potential regional difference in presynaptic mitochondrial availability. Indeed, several studies have demonstrated that the number of mitochondria-containing boutons is different between cortical regions and between cortical and subcortical areas in the mouse and human brains (de Vivo et al., 2017; Pickett et al., 2018; Petersen et al., 2019). These diverse synaptic alterations in the 5xFAD mPFC are summarized in Figure 4E. Collectively, our findings indicate that synapses devoid of presynaptic mitochondria are specifically decreased in layer 2 of the 5xFAD mPFC (Figure 3).

Although the precise mechanisms underlying the selective removal of synapses lacking mitochondria in 5xFAD mice remain to be addressed, studies have demonstrated that Aβ-induced disruption of presynaptic calcium clearance activates the phosphorylation of calcium/calmodulin-dependent protein kinase IV and synapsin 1. This impairs vesicle recycling and enhances aberrant neurotransmitter release, resulting in ultimate vesicle depletion (Parodi et al., 2010; Park et al., 2017). Thus, mitochondria-mediated local calcium homeostasis in presynaptic terminals may alleviate synaptic degeneration in AD. However, in the remaining mitochondria-containing synapses in the 5xFAD mPFC, we also observed a lower number of mitochondria per bouton (Figure 4), implying a putative eventual degeneration of mitochondria-containing synapses, potentially due to excessive oxidative stress at later stages of AD (De Felice et al., 2007). It is worth noting that we did not assess alterations in cristae structure of synaptic mitochondria since cristae membranes were not reliably differentiated in the SB-SEM images, possibly due to the strong over-staining with heavy metals. Future work should investigate the time course of changes in mitochondrial properties, including cristae structure, membrane potential, superoxide production, and gene expression profiles in AD mouse models using various functional assays and imaging modalities.

In the mature brain, thin spines with small PSDs exhibit rapid turnover, whereas mushroom spines with larger PSDs and a higher density of AMPA-type glutamate receptors persist for at least several months (Bourne and Harris, 2007). Thus, stable mushroom spines have been considered to act as ‘memory' spines, whereas dynamic thin spines capable of converting to mushroom spines upon strong stimulation are thought to act as ‘learning' spines (Holtmaat et al., 2005; Bourne and Harris, 2007). In this study, we found a selective reduction in less-stable thin spines in the 5xFAD mPFC (Figure 3). Interestingly, an APP transgenic mouse line displayed impaired maintenance of spine density through increased elimination of rapidly changing spines (Spires-Jones et al., 2007), although they did not classify these dynamic spines according to shape. In support of this view, our data add a novel finding that the majority of thin spines contact presynaptic boutons lacking mitochondria (Figure 3). Thus, both the dynamic nature and absence of presynaptic mitochondria in thin spines may contribute to the enhanced susceptibility of this spine type to amyloid pathology. We speculate that the preferential loss of thin spines in the 5xFAD mPFC might be related with cognitive impairments in AD; i.e., deficits in recent memories at early stages preceding the loss of long-term memory at later stages.

In conclusion, our findings highlight a selective loss of synapses lacking presynaptic mitochondria and a reduced number of mitochondria per presynaptic bouton in the 5xFAD mPFC. Our results suggest region- and type-specific vulnerability of cortical synapses in AD and that mitochondria in presynaptic terminals may affect cortical synaptic degeneration in AD.

## Data Availability Statement

The original contributions presented in the study are included in the article/Supplementary Material, further inquiries can be directed to the corresponding author.

## Ethics Statement

The animal study was reviewed and approved by The Korea Brain Research Institute (KBRI) Institutional Animal Care and Use Committee (IACUC-19-00033 and IACUC-20-00040).

## Author Contributions

KJL designed the experiments, wrote the manuscript, and supervised the project. N-yS, GHK, JEN, JWS, and CHL performed the experiments and analyzed the data. N-yS wrote the initial draft of the manuscript. All authors contributed to the article and approved the submitted version.

## Conflict of Interest

The authors declare that the research was conducted in the absence of any commercial or financial relationships that could be construed as a potential conflict of interest.
