# Supplementary material for: Selective Regional Loss of Cortical Synapses Lacking Presynaptic Mitochondria in the 5xFAD Mouse Model
Source: Front Neuroanat. 2021 Jun 25;15:690168. doi: 10.3389/fnana.2021.690168 (PMC8267061; doi:10.3389/fnana.2021.690168)
Supplement: Supplementary file 1 [file Table_1.pdf]

Table 1. Summary of the quantified parameters in the study.

Parameters	WT_mPFC Mean±SEM	n	5xFAD_mPFC Mean±SEM	n	WT_V1 Mean±SEM	n	5xFAD_V1 Mean±SEM	n
**Immunostaining using anti-6E10**								
Average size of puncta (μm²)								
L1	15.62 ± 4.89	5	209.36 ± 37.13	5	45.10 ± 20.17	5	55.59 ± 15.28	5
L2-3	0.00 ± 0.00	5	215.37 ± 28.32	5	17.85 ± 5.14	5	41.27 ± 10.09	5
L4	NA		NA		8.92 ± 3.99	5	65.82 ± 5.01	5
L5-6	12.27 ± 3.38	5	651.14 ± 41.80	5	0.00 ± 0.00	5	453.96 ± 27.82	5
Number of puncta / mm²								
L1	0.93 ± 0.26	5	10.68 ± 0.84	5	6.56 ± 2.93	5	5.35 ± 1.47	5
L2-3	0.00 ± 0.00	5	20.87 ± 3.00	5	0.53 ± 0.17	5	2.52 ± 0.51	5
L4	NA		NA		0.32 ± 0.14	5	2.93 ± 0.35	5
L5-6	0.31 ± 0.09	5	60.11 ± 5.64	5	0.00 ± 0.00	5	38.19 ± 4.94	5
**Electron Microscopy**								
Dendrite regularity index	0.24 ± 0.009	12	0.17 ± 0.005	12	0.40 ± 0.010	9	0.39 ± 0.009	9
Spine density (# / 10μm)	16.27 ± 1.35	12	12.64 ± 0.76	12	17.27 ± 0.83	9	16.14 ± 1.49	9
Synapse density (# / 10μm)	14.57 ± 1.14	12	10.96 ± 0.59	12	14.28 ± 1.09	9	13.00 ± 1.02	9
Density without presynaptic mitochondria (#/10μm)	8.99 ± 1.05	12	6.17 ± 0.44	12	9.59 ± 0.66	9	8.94 ± 0.76	9
Density with presynaptic mitochondria (#/10μm)	5.30 ± 0.44	12	4.74 ± 0.50	12	4.69 ± 0.68	9	4.06 ± 0.41	9
Synapse density with multiple mitochondria (#/10μm)	1.91 ± 0.22	12	0.93 ± 0.20	12	0.43 ± 0.15	9	0.65 ± 0.22	9
**Mitochondria # / bouton**	1.44 ± 0.007	96	1.23 ± 0.006	87	1.14 ± 0.007	65	1.18 ± 0.007	61
**Mitochondrial volume (μm³)**	0.04 ± 0.0002	138	0.05 ± 0.0002	106	0.05 ± 0.0004	74	0.05 ± 0.0005	71
Spine volume (μm³)								
Spine volume	0.09 ± 0.0003	289	0.10 ± 0.0005	228	0.09 ± 0.0003	239	0.09 ± 0.0004	238
Spine volume without presynaptic mitochondria	0.06 ± 0.0003	154	0.06 ± 0.0005	104	0.08 ± 0.0005	134	0.08 ± 0.0005	132
Spine volume with presynaptic mitochondria	0.15 ± 0.0014	97	0.18 ± 0.0016	85	0.14 ± 0.0014	65	0.17 ± 0.0021	61
Axon-spine interface (ASI) area (μm²)								
ASI area	0.14 ± 0.0008	251	0.15 ± 0.0010	188	0.18 ± 0.0011	189	0.23 ± 0.0015	185
ASI area without presynaptic mitochondria	0.08 ± 0.0007	154	0.07 ± 0.0009	100	0.12 ± 0.0010	126	0.15 ± 0.0013	123
ASI area with presynaptic mitochondria	0.24 ± 0.0026	95	0.24 ± 0.0025	87	0.29 ± 0.0041	63	0.38 ± 0.0060	61
Spine neck diameter (μm)								
Neck diameter (total)	0.22 ± 0.0003	274	0.21 ± 0.0004	215	0.26 ± 0.0004	238	0.25 ± 0.0004	236
Neck diameter without presynaptic mitochondria	0.21 ± 0.0006	146	0.21 ± 0.0007	101	0.26 ± 0.0007	133	0.25 ± 0.0006	131
Neck diameter with presynaptic mitochondria	0.24 ± 0.0009	93	0.24 ± 0.0013	76	0.29 ± 0.0015	65	0.29 ± 0.0018	61
Spine head diameter (μm)								
Head diameter (total)	0.44 ± 0.0007	274	0.46 ± 0.0011	215	0.46 ± 0.0008	238	0.47 ± 0.0009	236
Head diameter without presynaptic mitochondria	0.40 ± 0.0010	146	0.40 ± 0.0012	101	0.45 ± 0.0013	133	0.46 ± 0.0014	131
Head diameter with presynaptic mitochondria	0.57 ± 0.0026	93	0.66 ± 0.0035	76	0.59 ± 0.0027	65	0.63 ± 0.0038	61
Spine shape classification								
Thin type (# / 10μm)	7.50 ± 0.83	12	5.00 ± 0.72	12	9.91 ± 0.69	9	8.52 ± 0.63	9
Mushroom type (# / 10μm)	7.01 ± 0.77	12	6.28 ± 0.45	12	6.07 ± 0.93	9	6.20 ± 0.95	9
Stubby type (# / 10μm)	0.44 ± 0.22	12	0.38 ± 0.13	12	1.23 ± 0.28	9	1.12 ± 0.44	9
Branched type (# / 10μm)	0.88 ± 0.23	12	0.71 ± 0.23	12	0.14 ± 0.09	9	0.15 ± 0.10	9
Filopodia type (# / 10μm)	0.06 ± 0.06	12	0.05 ± 0.05	12	0.00 ± 0.00	9	0.07 ± 0.07	9
Relative distribution of presynaptic partner								
Thin spine-contacting boutons lacking mitochondria	0.69 ± 0.02	12	0.63 ± 0.03	12	0.78 ± 0.01	9	0.81 ± 0.01	9
Thin spine-contacting boutons with mitochondria	0.31 ± 0.02	12	0.37 ± 0.03	12	0.22 ± 0.01	9	0.19 ± 0.01	9
Mushroom-contacting boutons lacking mitochondria	0.48 ± 0.02	12	0.45 ± 0.01	12	0.54 ± 0.02	9	0.52 ± 0.02	9
Mushroom-contacting boutons with mitochondria	0.52 ± 0.02	12	0.55 ± 0.01	12	0.46 ± 0.02	9	0.48 ± 0.02	9
