# Supplementary material for: Selective Regional Loss of Cortical Synapses Lacking Presynaptic Mitochondria in the 5xFAD Mouse Model
Source: Front Neuroanat. 2021 Jun 25;15:690168. doi: 10.3389/fnana.2021.690168 (PMC8267061; doi:10.3389/fnana.2021.690168)
Supplement: Supplementary file 2 [file Image_1.pdf]

Supplemental Figures

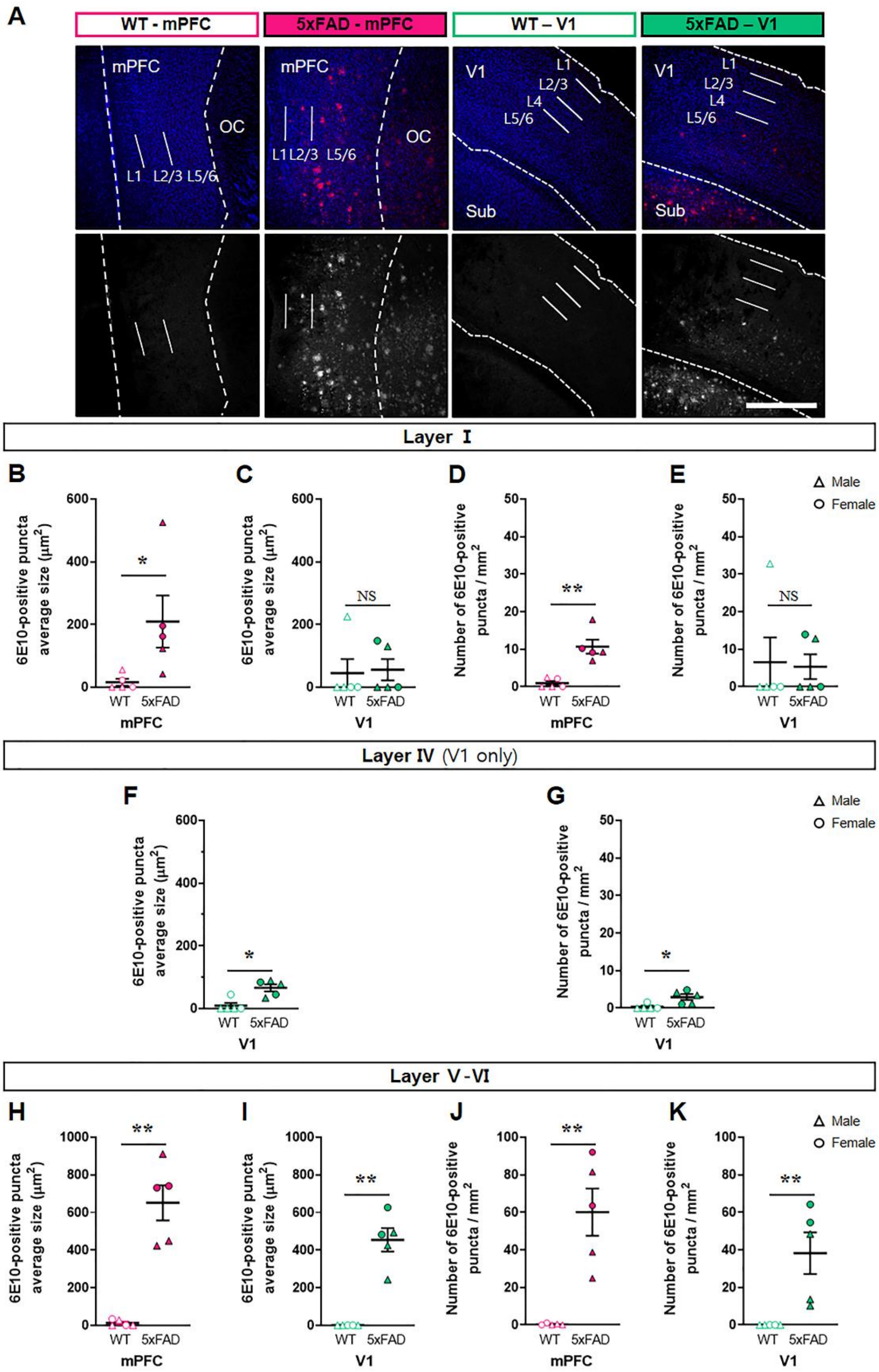

Figure S1. Layer-specific amyloid accumulation in the 5xFAD cortex.

(A) Representative fluorescence images of amyloid plaques stained with 6E10 antibody in the mPFC and V1 of wild-type (WT) and 5xFAD mice. Note that mPFC lacks layer 4. Dashed Lines indicate the cortical borders and solid lines indicate the quantified cortical layers. mPFC,

medial prefrontal cortex; V1, primary visual cortex. Scale bars, 500 µm. (B-E) Quantification of amyloid puncta size (B and C) and puncta density (D and E) in layer 1 of the mPFC and V1, respectively (n=5 animals per group). (Layer 1 [puncta size, µm^2]: mPFC, $P = 0.02$; V1, $P > 0.99$; [puncta #/mm^2]: mPFC, $P = 0.008$; V1, $P > 0.99$; Mann–Whitney U test). (F and G) Quantification of amyloid puncta size (F) and puncta density (G) in layer 4 of V1 (n=5 animals per group). (Layer 4 [puncta size, µm^2]: V1, $P = 0.02$; [puncta #/mm^2]: V1, $P = 0.02$; Mann–Whitney U test). (H-K) Quantification of amyloid puncta size (H and I) and puncta density (J and K) in layer 5/6 of the mPFC and V1 (n=5 animals per group). (Layer 5/6 [puncta size, µm^2]: mPFC, $P = 0.008$; V1, $P = 0.008$; [puncta #/mm^2]: mPFC, $P = 0.008$; V1, $P = 0.008$; Mann–Whitney U test). *P<0.05, **P<0.01.

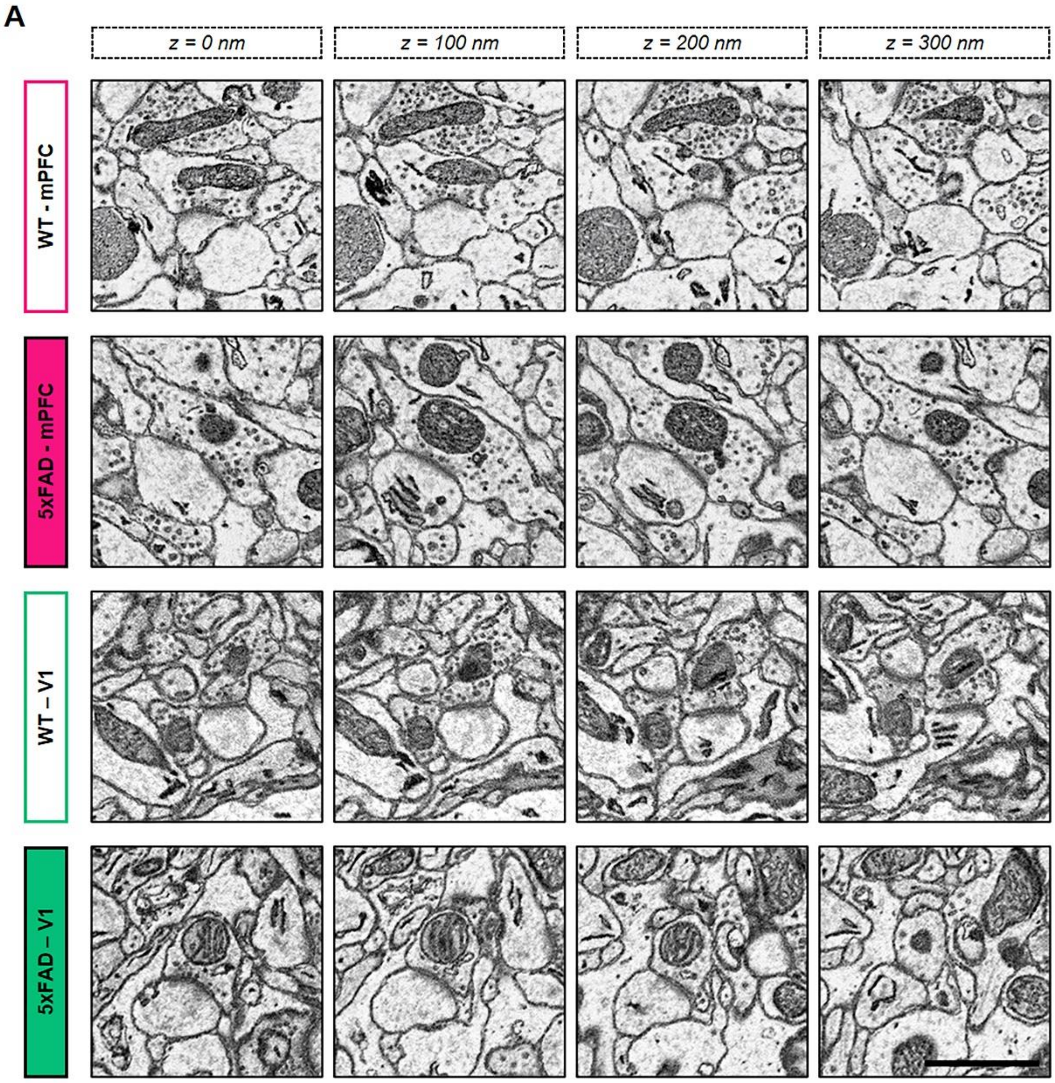

Figure S2. SB-SEM images acquired in the mPFC and V1 of WT and 5xFAD mice.

(A) Representative EM images of layer 2 synapses in the mPFC and V1 of WT and 5xFAD mice. Four serial images of the same synapse are shown for each genotype and region. Scale bars, 1 µm.

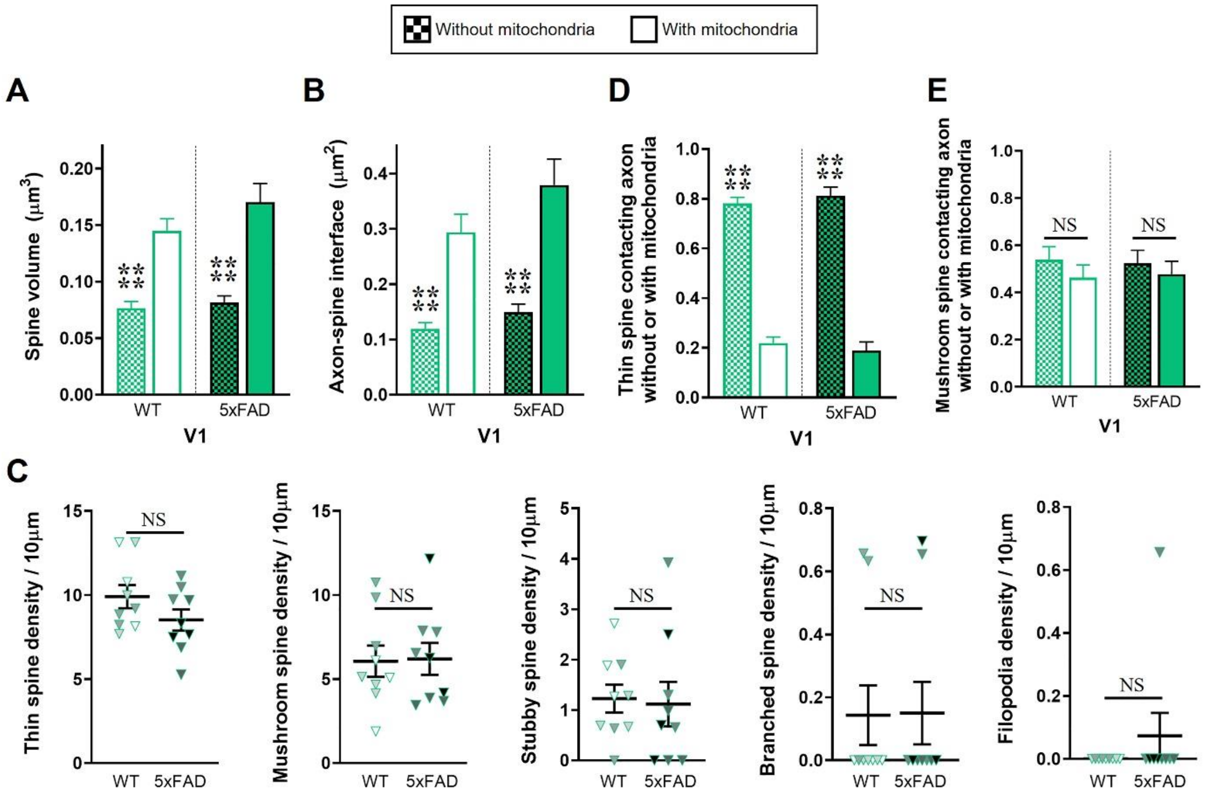

Figure S3. Morphological analysis of synapses and dendritic spines in the V1 of 6-month-old WT and 5xFAD mice.

(A and B) Quantification of average spine volume (A) and ASI area (B) of synapses with or without presynaptic mitochondria in the V1 of WT and 5xFAD mice (n=61–63 mitochondria-containing synapses; 123–126 mitochondria-lacking synapses). ****P<0.0001; Kruskal–Wallis test with post hoc Dunn test. (C) Quantification of the number of spines according to shape per 10 μm dendritic length (n=9 dendrites per group). ([spine #/10 μm]: thin, $P = 0.16$; mushroom, $P = 0.92$; stubby, $P = 0.84$; branched, $P = 0.96$; unpaired t-test; filopodia, $P > 0.99$; Mann–Whitney U test). (D) The proportion of thin spines contacting boutons with or without mitochondria in each dendritic segment (n=9 dendrites per group). ****P<0.0001; two-way ANOVA with post hoc Tukey test. (E) The proportion of mushroom spines contacting boutons with or without mitochondria in each dendritic segment (n=9 dendrites per group). (WT, $P = 0.73$; 5xFAD, $P = 0.94$; two-way ANOVA with post hoc Tukey test).
